# Clinical evidence for the use of aspirin in the treatment of cancer

**DOI:** 10.3332/ecancer.2013.297

**Published:** 2013-03-20

**Authors:** Ruth E Langley

**Affiliations:** MRC Clinical Trials Unit, Aviation House, 125 Kingsway, London WC2B 6NH, UK and; Brighton and Sussex University Hospitals Trust , Brighton, UK

**Keywords:** aspirin, cancer, treatment, toxicity, mechanisms

## Abstract

Although the anti-cancer effects of aspirin were first identified in pre-clinical models four decades ago, a clear role for the drug in either the prevention or treatment of cancer has not been established. Concerns about toxicity, particularly major haemorrhage, and a lack of randomised evidence demonstrating efficacy have limited its use in primary prevention; there was also doubt that a simple aspirin could have a significant therapeutic effect against established malignancy. Three new pieces of evidence: a series of meta-analyses focusing on cancer outcomes from randomised-controlled trials designed to assess the vascular benefits of daily aspirin; the first positive results from a randomised-controlled trial designed to demonstrate that aspirin can prevent cancer in those with a hereditary predisposition; and observational data showing that aspirin use after a cancer diagnosis improves both cancer mortality and overall survival; have led to a re-evaluation of aspirin as a potential anti-cancer agent both for the prevention and treatment of cancer.

## Introduction

Aspirin (acetylsalicylic acid) is used as an analgesic/anti-inflammatory and in the prevention of cardiovascular disease. The observation, over four decades ago, that agents which induce thrombocytopenia reduce metastases [1] led to the investigation of aspirin as an anticancer drug, with metastases from fibrosarcomas in animal models significantly reduced by aspirin [2]. In 1988, the first clinical study was published showing that regular aspirin use was associated with a significantly lower risk of developing colorectal cancer [3]. Subsequently, however, two large randomised placebo-controlled trials assessing alternate day aspirin as a primary prevention strategy, after a mean follow-up of ten years, reported no significant difference in cancer incidence or mortality [4, 5] and focus shifted away from aspirin to the related but newer selective cyclo-oxygenase (Cox)-2 inhibitors as potential anti-cancer agents. In 2005, concerns about previously unrecognised cardiac effects led to the withdrawal of some selective Cox-2 inhibitors and early closure of ongoing trials [6–8]. This review is aimed primarily at oncologists; it focuses on clinical research published over the last five years that has led to a re-evaluation of aspirin as a potential anti-cancer agent. Resulting insights into the biological mechanisms underlying the anti-cancer effects of aspirin and opportunities for employing aspirin in the field of oncology are discussed.

## Aspirin and cancer: recently published evidence

Over the last five years, Rothwell and colleagues have published a series of systematic reviews and individual patient data meta-analyses evaluating cancer outcomes from randomised-controlled trials designed to assess the vascular benefits of daily aspirin. The initial report [9] reviewed two large trials (*n *= 7588) that recruited patients in the United Kingdom between 1978 and 1985 assessing aspirin within a dose range of 300–1200 mg daily. Median follow-up was 23 years with cancer outcomes available from the United Kingdom National Cancer Registry. In this particular trial, participants who admitted to being non-compliant with allocated treatment were excluded. Aspirin use resulted in a significant reduction in colorectal cancer incidence; this was dependent on duration of treatment, compliance and length of follow-up, with the greatest effect seen 10–14 years after randomisation in those receiving scheduled treatment for five years or more (Hazard ratio (HR) 0.26 95% confidence interval (CI) 0.12–0.56 *P *= 0.0002). No significant effect on the incidence of other cancers was noted in this study.

Subsequent analyses, including six further trials (*n *= 25,570) with a mean duration of aspirin therapy of four years or more [10, 11], demonstrated that the protective effects of aspirin were also seen at lower doses (75–300 mg) and across tumour types, particularly those arising from the gastrointestinal tract. For example, the 20-year risk of cancer death from gastrointestinal cancer was reduced with a HR of 0.65 (95% CI 0.54–0.78) *P *< 0.0001 for those allocated to aspirin compared to non-users. The most recent data [12] have included 51 trials (77,000 participants) randomly assigned to daily aspirin versus no aspirin or other anti-platelet agent. Aspirin reduced the incidence of cancer, with most benefit seen when the scheduled duration of trial treatment was five years or more (HR 0.81 (95% CI 0.70–0.93) *P *= 0.003), and resulted in a relative reduction in cancer deaths of about 15% (Table 1). Stratification by time to death identified positive effects on cancer mortality as early as three years from randomisation, particularly in trials employing high-dose aspirin (>300 mg daily). This early effect raised the possibility that aspirin could be inhibiting the growth and development of metastases from previously unrecognised cancers, and this was supported by subsequent data showing that allocation to aspirin reduced the risk of metastases at diagnosis (HR 0.69 (95% CI 0.50–0.95) *P *= 0.02) and the subsequent risk of developing metastases (HR 0.45 (95% CI 0.28–0.72) *P *= 0.0009) [13]. These latter findings, in particular, provide compelling evidence for the use of aspirin as an adjuvant agent in the treatment of cancer.

**Figure 1:**
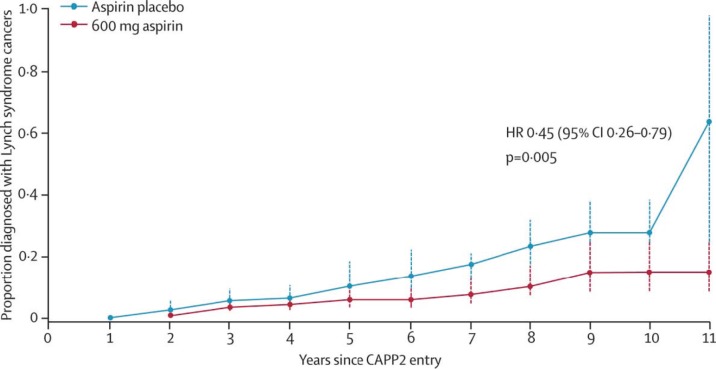
Recent evidence confirming the anti-cancer effects of aspirin. Proportion of patients developing a Lynch Syndrome related cancer over time in the randomised-placebo controlled CAPP2 trial [14]. Participants received aspirin (600 mg daily) or a placebo. with the analysis restricted to those who took trial treatment for 2 years or more. From Burn J et al (2012) Long-term effect of aspirin on cancer risk in carriers of hereditary colorectal cancer: an analysis from the CAPP2 randomised controlled trial Lancet 378 (9809), 2081-7. Reprinted with kind permission from Elsevier.

The results of these meta-analyses have been reinforced by long-term data from the randomised CAPP2 trial [14]. In this study, 861 participants with Lynch syndrome (hereditary non-polyposis colon cancer), who carry a germline mutation in DNA mismatch repair enzymes resulting in a predisposition to developing colorectal and other cancers, were assigned to aspirin (600 mg daily) or a placebo. After a mean duration of treatment of 25 months and follow-up of 55 months, the time to first colorectal cancer was significantly reduced for those taking aspirin (HR 0.63 (95% CI 0.35–1.13) *P *= 0.12), with an incidence rate ratio (IRR) of 0·56 (95% CI 0.32–0.99) *P *= 0.05. The results were more marked for those who completed at least two years of treatment, and there was also evidence of a reduced incidence of other tumours associated with Lynch syndrome, including endometrial, ovarian, pancreatic, brain, small bowel, gall bladder, ureter, stomach and kidney cancer (Figure 1). Aspirin is now considered a standard of care for this group, and a subsequent study is planned to assess whether lower doses of aspirin will be equally effective.

Adding to this evidence is accumulating non-randomised data on aspirin use after a cancer diagnosis (Table 2). The Nurses Health Study is a prospective cohort of over 120,000 female nurses in the United States; 4164 were diagnosed with stage I–III breast cancer between 1976 and 2002 and followed up until death or 2006. Aspirin use after diagnosis, assessed every two years as part of a predetermined questionnaire, was associated with a decreased risk of breast cancer death. The adjusted relative risks (RR) for 1, 2–5 and 6–7 days of aspirin use per week (primarily 325 mg daily) compared with no use were 1.07 (95% CI 0.70–1.63), 0.29 (95% CI 0.16–0.52) and 0.36 (95% CI 0.24–0.54), respectively (test for linear trend, *P *< 0.001) [15]. This association did not differ appreciably by stage, menopausal status, body mass index or oestrogen receptor status. Similar results have been seen for colorectal cancer, in the Nurses Health Study, and the Health Professionals Follow-Up Study where aspirin use after a diagnosis of colorectal cancer was associated with a significant reduction in colorectal cancer deaths (adjusted HR 0.71 (95% CI 0.53–0.95) as well as overall mortality (adjusted HR 0.79 (95% CI 0.65–0.97), with larger effects observed for daily users [16]. The beneficial effects on colorectal cancer mortality have also been confirmed in contemporary cohorts receiving low-dose aspirin (75–80 mg daily). For example, a large Dutch population-based study has shown a reduction in overall mortality associated with aspirin use following a colon cancer diagnosis (adjusted RR 0.65 (95% CI 0.50–0.84) *P *= 0.001) [17], and similar results have been observed in an audit of colorectal cancer patients in the United Kingdom with an adjusted HR of 0.67 (95% CI 0.57–0.79) *P *< 0.001 for all cause mortality [18]. Strengths of these latter studies include encashed prescribing, rather than patient recall to determine aspirin usage and more precise information about dose.

In prostate cancer, aspirin use after radical treatment (either surgery or radiotherapy) has been shown to decrease biochemical failure and prostate-cancer-specific mortality. In a retrospective multivariate analysis of 2051 men who had undergone radical radiotherapy for carcinoma of the prostate, aspirin non-use was associated with early biochemical failure (odds ratio (OR) 2.05 (95% CI 1.33–3.17) *P *= 0.0012) [19], and recent data from the CAPSURE registry on approximately 6000 men who had undergone either radical surgery or radiotherapy shows that anticoagulant use (mainly aspirin) reduced the risk of prostate cancer mortality from 8% in the control group to 3% in those receiving anticoagulants *P *< 0.01. The risks of disease recurrence and bone metastasis were also significantly lower, and the reduction in prostatecancer-specific mortality was most marked in those with a high risk of disease recurrence (19% versus 4% at ten years *P *< 0.01) [20]. In gastro-oesophageal cancer, early data from a Chinese study suggests that taking aspirin after an oesophagectomy for either squamous cell carcinoma or adenocarcinoma is associated with improved outcomes, with five-year survival rates of 51.2% for those who received aspirin (*n* = 445) compared to placebo 41% (*n* = 658) or no tablet 42.3% (*n* = 495) *P* = 0.04 [21].

## Overview of other evidence

The data described above all support the use of aspirin as an anti-cancer agent, but the results of previous studies have been less consistent—in particular, data published to date from the Physicians Health Study, a large randomised placebo-controlled 2 × 2 factorial trial evaluating aspirin (325 mg on alternative days) for cardiovascular protection and beta carotene as a primary prevention agent against colorectal cancer that recruited over 22,0000 male physicians (40–84 years of age) between 1982 and 1988. The aspirin component of the study was terminated early, after a mean follow-up of five years, because of a statistically significant reduction in first myocardial infarction (RR 0.56 (95% CI 0.45–0.70) *P *< 0.00001); participants were unblinded and allowed to self select aspirin use. Even with long-term follow-up (>12 years), no effect on colorectal cancer incidence (RR 1.03 (95% CI 0.83–1.28)) was found in those randomly assigned to aspirin [4]. A similar result was initially reported in the Women’s Health Study, where approximately 40,000 women over the age of 45 in the United States without a history of cancer, cardiovascular disease, or other major chronic illness were randomised to placebo or aspirin (100 mg) on alternate days. After a mean follow-up of ten years, no effect of aspirin was observed on total cancer incidence (RR 1.01 (95% CI 0.94–1.08) *P *= 0.87) nor any specific cancer incidence, with the exception of lung cancer, where a trend towards reduction of risk (RR 0.78 (95% CI 0.59–1.03) *P *= 0.08) was observed; there was also no reduction in cancer mortality (RR 0.95 (95% CI 0.81–1.11) [5]. This led the United States preventative services task force in 2007 to recommend that aspirin and other non-steroidal anti-inflammatory drugs (NSAID) were not prescribed to individuals at average risk of developing colorectal cancer as a primary prevention strategy [22]. However, it has been recently reported that during a seven-year extension of the Women’s Health Study that the incidence of colorectal cancer was 18% lower in the group receiving aspirin, with the effect only emerging after ten years [23].The difficulties in remembering to self-administer an alternate day therapy, particularly in the primary prevention setting, may have led to poorer compliance in these studies and may partly explain the differences in outcomes compared to the more recent data from the vascular studies assessing daily aspirin use.

Further supporting evidence for the use of aspirin as an anti-cancer drug include 194 cohort and case–control studies, recently reviewed by Bosetti [24], showing consistent and significant reductions in cancer incidence across a number of common solid tumours including colorectal, breast, gastro-oesophageal (both adenocarcinomas and squamous cell), lung and prostate cancer in those that regularly use aspirin (see Table 3). In addition, a meta-analysis of secondary prevention studies in those with previous colorectal cancer or adenoma estimates that aspirin reduces the RR of further adenomas by 18% (RR 0.82 (95% CI: 0.74–0.91) *P *= 0.0002) [25]. There are also three small randomised studies, reviewed previously [26], that added aspirin to standard chemotherapy regimens in the advanced setting in the late 1980s that appear to show no effect on cancer outcomes. However, the magnitude of difference the studies were designed to detect was ambitious and as such should not lead to the definitive conclusion that aspirin is ineffective in the advanced setting, though demonstrating efficacy in this setting with a drug whose anti-cancer effects may well be at the earliest stage of both primary tumour and metastases formation will be challenging.

## Mechanism of action

The recent data have renewed interest in the potential mechanisms of action underlying the anti-cancer effects of aspirin. Aspirin inhibits the enzyme Cox, and many of the downstream mediators of this pathway are thought to be involved in the development and spread of malignancy [27]. However, aspirin has a short half-life (approximately 20 min), and although it irreversibly inactivates Cox-1 and Cox-2 through selective acetylation, nucleated cells can resynthesise Cox isozymes within a few hours. Thus, a single daily dose of aspirin (75–100 mg), as used in the contemporary vascular studies analysed by Rothwell [10–12] and the more recent observational cohort studies [18, 17], is unlikely to have a direct effect on Cox pathways in systemic tissues. To achieve consistent inhibition of Cox in tissues, an analgesic dose of aspirin, e.g., a divided daily dose of >2000 mg of aspirin, would be required [28].

A once daily dose of aspirin (75–100 mg) is considered to have negligible direct biological effects apart from on the anucleate platelet through inhibition of Cox-1. Platelets are thought to effect the development and spread of metastases by facilitating the adhesion of cancer cells to circulating leukocytes and endothelial cells and permitting adhesion to the endothelium and transmigration [29].They are also thought to protect circulating cancer cells from immune-mediated clearance by natural killer cells [30]. Recent evidence also suggests that platelets may play a more active role in promoting metastatic spread outside of the primary tumour’s microenvironment by active signalling to tumour cells through the TGF-B and NF-Kb pathways resulting in a pro-metastatic phenotype that facilitates tumour cell extravasation and metastasis formation [31], and aspirin is known to inhibit the activation of NF-Kb [32].

There is also a significant body of evidence indicating that selective Cox-2 inhibitors could also be useful anti-cancer agents, and they have been shown to prevent adenoma formation in randomised trials [33]. The rationale for evaluating selective Cox-2 inhibitors as anti-cancer agents came from two strands; first epidemiological and in vitro studies had shown that aspirin and other NSAID agents had anti-cancer effects [34], and it was presumed that, given that both aspirin and NSAIDs inhibit Cox, this was the significant and predominant mechanism of action underlying the anti-cancer effects; second, increased Cox-2 expression was demonstrated in a number of different tumour types and associated with poor prognosis [35] with mechanistic hypotheses linking Cox-2 inhibition to apoptotic and anti-angiogenic pathways, which are thought to be key to the prevention and treatment of cancer [36].

Patrono has suggested that inhibition of Cox-1 in platelets by low-dose aspirin suppresses the induction of Cox-2 in distant nucleated cells within the tumour or stromal environment in the early stages of neoplasia [28]. At sites of intestinal mucosal injury, platelets trigger downstream signalling events leading to reduced apoptosis, enhanced cellular proliferation and angiogenesis, which can be indirectly inhibited by aspirin. This would explain the observations that both daily low-dose aspirin and selective Cox-2 inhibitors appear to be effective anticancer drugs and is supported by studies showing that inhibition of either Cox-1 or Cox-2 is sufficient to inhibit tumourigenesis in mouse models [37], and from experiments with mice modified genetically to be deficient in either Cox-1 or Cox-2. Despite Cox-1 being considered a “housekeeping” enzyme constitutively expressed in most tissues, mice deficient in Cox-1, with prostaglandin levels reduced by 99% in most tissues, have normal pathology in both the kidney and stomach, and do not develop gastric ulcers. In contrast, Cox-2 deficient mice have reduced life spans, with 40% dying before weaning from unknown causes [38]. Of particular relevance is how these mice have been used to study the role of Cox in malignant transformation. Mice with a chemically induced mutation in the Apc gene, which results in 100% of the mice having intestinal neoplasia, were also made Cox-1 or Cox-2 deficient. Both Cox-1/Apc and Cox-2/Apc deficient mice showed an 80% decrease in intestinal polyps indicating that inhibition of either Cox-1 or Cox-2 could be an effective anti-cancer strategy [39].

The recent observation [40] that the improvements in cancer and overall mortality seen in two large prospective cohorts associated with aspirin use after a diagnosis of colorectal cancer correlate with the presence of mutated PIK3CA within the tumour provides further insights into the possible anti-cancer mechanisms of aspirin, as well as raising the possibility that molecular profiling may be able to select patients most likely to respond to aspirin. Although some caution is required when interpreting these data as they were non-randomised and the number of patients with mutated *PIK3CA *who regularly used aspirin was small (*n *= 66), the results are striking, with a multivariate HR for cancer death of 0.18 (95% CI 0.06–0.61) *P *< 0.001 and 0.54 (95% CI 0.31–0.94) *P *= 0.01 for death from any cause for those that regularly took aspirin after a diagnosis of colorectal cancer. Given the relatively low frequency of *PIK3CA *mutations (10–20%) in colorectal cancer, it is unlikely that an effect on the mutated *PIK3CA *tumours alone could explain the large effects of aspirin on colorectal cancer incidence and mortality observed in the randomised vascular trials [10].

From a clinical perspective, as the initial mechanism of action of daily low-dose aspirin (primarily on platelets) and that of selective Cox-2 inhibitors is different, results from studies evaluating the anti-cancer effects of these drugs cannot be assumed to be identical. In addition, the evidence suggests that aspirin (and possibly selective Cox-2 inhibitors) are most likely to be effective anti-cancer agents when the tumour burden is low, i.e., in the primary prevention or adjuvant setting. Thus, results from randomised trials assessing selective Cox-2 inhibitors in more advanced disease, such as the STAMPEDE trial [41] or ongoing studies evaluating selective Cox-2 inhibitors in the adjuvant setting, e.g., the REACT trial (ISRCTN48254013) in breast cancer, should not prevent the development of randomised studies evaluating aspirin particularly in the adjuvant setting.

## Toxicity

The use of aspirin in the prevention and treatment of cancer to date has been limited by concerns about toxicity, particularly, serious haemorrhage. Data available from the Antithrombotic Trialists’ (ATT) Collaboration [42] in a meta-analysis of six primary prevention studies (*n *= 95,456, demographics: mean age 56 years, 46% men, mean cholesterol 5.6 mmol/L, 41% hypertensive, 4% diabetic and 16% current smokers) found allocation to aspirin increased the incidence of gastrointestinal haemorrhage or other serious extracranial bleed from 0.07% to 0.1% per year, HR 1.54 (95% CI 1.30–1.82) *P *< 0.0001. The increased risk was mainly non-fatal bleeds, and there were fewer fatal bleeds in participants allocated aspirin compared to the controls. Although this may have occurred by chance, a similar pattern was seen in the recent meta-analyses performed by Rothwell. Allocation to aspirin increased major extracranial haemorrhage in the first three years, OR 1.95 (95% CI 1.47–2.59) *P *< 0.0001, but from three years onward the OR decreased to 1.04 (95% CI 0.73–1.49) *P *= 0.90 with the proportion of bleeds that were fatal being lower on aspirin than control, (15/132 placebo vs. 8/203 aspirin) OR 0.32 (95% CI 0.12–0.83) *P *= 0.009, and this difference remained when the analysis was restricted to individuals who were still on allocated trial treatment at the time of the bleed [12]. Until recently, policy makers evaluating the use of aspirin have primarily considered vascular benefits versus concerns about serious haemorrhage; however, even a modest effect of aspirin on cancer outcomes, e.g. a 10% reduction in overall cancer incidence will alter the risk/benefit ratio and broaden the population most likely to benefit from aspirin [28].

## Conclusion

In summary, data from randomised clinical studies have shown that aspirin prevents the development of malignancy, and it also appears to decrease the development and spread of metastases. There may be at least two separate mechanisms underlying these effects: first, an anti-platelet effect that is predominantly responsible for the effect on the dissemination of blood-born metastases; and second, an effect on Cox-2 pathways (potentially mediated through platelets again) that affects the primary development of the tumour and possibly the early growth of metastases. These hypotheses need to be tested in randomised trials, and they have implications not only for the design of studies evaluating aspirin but also for those evaluating selective Cox-2 inhibitors and other anti-platelet drugs that may have similar mechanisms of action to aspirin.

## Figures and Tables

**Table 1:** Recent evidence confirming the anti-cancer effects of aspirin. Cancer deaths from a meta-analysis of 51 randomised trials designed primarily to assess vascular outcomes where participants were assigned to daily aspirin versus no aspirin or other anti-platelet agent (*n *= 77,000) [12]. The outcome was not affected by aspirin dose or whether aspirin was administered for primary or secondary vascular prevention.

Follow up (years)	Aspirin – cancer deaths	Control – cancer deaths	Odds Ratio (95% CI)	P
0-2.9 years	292	325	0.90 (0.76 – 1.06)	0.18
3.0–4.9 years	161	173	0.93 (0.75 – 1.16)	0.51
>5 years	92	145	0.63 (0.49 – 0.82)	0.0005
Unknown	17	21		
**Total**	**562**	**664**	**0.85 (0.76 – 0.96)**	**0.008**

**Table 2:** Recent evidence confirming the anti-cancer effects of aspirin. Summary of observational data assessing the effect of aspirin after a cancer diagnosis by tumour type. *OS* overall survival, *HR* hazard ratio, *RR* relative risk, estimates given with 95% confidence intervals, $ data not available for OS, ^*^ data not available for cancer specific mortality

Tumour	Study/Year/Reference	Result (in favour of aspirin)
**Colorectal (CRC)**	Chan 2009 [16]	CRC mortality HR 0.71 (0.53–0.95) OS HR 0.79 (0.65-0.97)
	Bastiaannet 2012 [17]	OS RR 0.65 (0.50 - 0.84)
	McCowan 2012 [18]	CRC mortality HR 0.67 (0.57-0.79) OS HR 0.58 (0.45-0.75)
**Breast (BC)**	Holmes 2010 [15]	BC mortality RR 0.36 (0.24–0.65) OS RR 0.54 (0.41-0.70)
**Prostate (PC)**	Zaorsky 2012 [19]	Reduced interval to biochemical failure – aspirin non-use OR 2.05 (1.33-3.17)
	Choe 2012 [20]	PC mortality HR 0.43 (0.21-0.87)$
**Gastric Oesophagus**	Liu 2009 [21]	5 year OS aspirin 51.2%, placebo 41%, no tablet 42.3%^*^

**Table 3.** Summary of relative risks of developing cancer in regular aspirin users (at least 1-2 tablets per week) compared to non-users in several common solid tumours. Data from Bosetti *et al*. 2012 [24]—a meta-analysis of observational studies assessing aspirin use and cancer incidence. Data on pancreatic, endometrial, ovarian, bladder, and renal cancer are also available, but for these tumour types, the overall results did not reach statistical significance.

Cancer Type/Study	No of Studies	No of Cases	RR (95% CI)
**Colorectal**			
Case-control	15	21,414	0.63 (0.56-0.70)
Cohort	15	16,105	0.82 (0.75-0.89)
Overall	30	37,519	**0.73 (0.67-0.79)**
**Gastric Cancer**			
Case-control	7	2411	0.60 (0.44-0.82)
Cohort	6	2108	0.77 (0.58-1.04)
Overall	13	4519	**0.67 (0.54-0.83)**
**Oesophageal/cardia adenocarcinoma**			
Case-control	9	3222	0.60 (0.48-0.75)
Cohort	2	499	0.88 (0.68-1.15)
Overall	11	3721	**0.64 (0.52-0.78)**
**Oesophageal Squamous Cell Carcinoma/unknown**			
Case-control	7	1075	0.54 (0.44-0.67)
Cohort	4	1118	0.73 (0.51-1.07)
Overall	11	2193	**0.61 (0.50-0.76)**
**Breast**			
Case-control	10	28.835	0.83 (0.76-0.91)
Cohort	22	27,091	0.93 (0.87-1.00)
Overall	32	52.926	**0.90 (0.85-0.95)**
**Prostate**			
Case-control	9	5795	0.87 (0.74-1.02)
Cohort	15	31,657	0.91 (0.85-0.97)
Overall	24	37,452	**0.90 (0.85-0.96)**
**Lung**			
Case-control	5	4863	0.73 (0.55-0.98)
Cohort	15	11,356	0.98 (0.92-1.05)
Overall	20	16,219	**0.91 (0.84-0.99)**

## References

[1] Gasic GJ, Gasic TB, Stewart CC (1968). Antimetastatic effects associated with platelet reduction. Proc Natl Acad Sci USA.

[2] Gasic GJ, Gasic TB, Murphy S (1972). Anti-metastatic effect of aspirin. Lancet.

[3] Kune GA, Kune S, Watson LF (1988). Colorectal cancer risk, chronic illnesses, operations, and medications: case control results from the Melbourne Colorectal Cancer Study. Cancer Res.

[4] Sturmer T, Glynn RJ, Lee IM, Manson JE, Buring JE, Hennekens CH (1998). Aspirin use and colorectal cancer: post-trial follow-up data from the Physicians’ Health Study. Ann Intern Med.

[5] Cook NR, Lee IM, Gaziano JM, Gordon D, Ridker PM, Manson JE, Hennekens CH, Buring JE (2005). Low-dose aspirin in the primary prevention of cancer: the Women’s Health Study: a randomised controlled trial. JAMA.

[6] Bresalier RS, Sandler RS, Quan H, Bolognese JA, Oxenius B, Horgan K, Lines C, Riddell R, Morton D, Lanas A, Konstam MA, Baron JA (2005). Cardiovascular events associated with rofecoxib in a colorectal adenoma chemoprevention trial. N Engl J Med.

[7] Nussmeier NA, Whelton AA, Brown MT, Langford RM, Hoeft A, Parlow JL, Boyce SW, Verburg KM (2005). Complications of the COX-2 inhibitors parecoxib and valdecoxib after cardiac surgery. N Engl J Med.

[8] Solomon SD, McMurray JJ, Pfeffer MA, Wittes J, Fowler R, Finn P, Anderson WF, Zauber A, Hawk E, Bertagnolli M (2005). Cardiovascular risk associated with celecoxib in a clinical trial for colorectal adenoma prevention. N Engl J Med.

[9] Flossmann E, Rothwell PM (2007). Effect of aspirin on long-term risk of colorectal cancer: consistent evidence from randomised and observational studies. Lancet.

[10] Rothwell PM, Wilson M, Elwin CE, Norrving B, Algra A, Warlow CP, Meade TW (2010). Long-term effect of aspirin on colorectal cancer incidence and mortality: 20-year follow-up of five randomised trials. Lancet.

[11] Rothwell PM, Fowkes FG, Belch JF, Ogawa H, Warlow CP, Meade TW (2011). Effect of daily aspirin on long-term risk of death due to cancer: analysis of individual patient data from randomised trials. Lancet.

[12] Rothwell PM, Price JF, Fowkes FG, Zanchetti A, Roncaglioni MC, Tognoni G, Lee R, Belch JF, Wilson M, Mehta Z, Meade TW (2012). Short-term effects of daily aspirin on cancer incidence, mortality, and non-vascular death: analysis of the time course of risks and benefits in 51 randomised controlled trials. Lancet.

[13] Rothwell PM, Wilson M, Price JF, Belch JF, Meade TW, Mehta Z (2012). Effect of daily aspirin on risk of cancer metastasis: a study of incident cancers during randomised controlled trials. Lancet.

[14] Burn J, Gerdes AM, Macrae F, Mecklin JP, Moeslein G, Olschwang S, Eccles D, Evans DG, Maher ER, Bertario L, Bisgaard ML, Dunlop MG, Ho JW, Hodgson SV, Lindblom A, Lubinski J, Morrison PJ, Murday V, Ramesar R, Side L, Scott RJ, Thomas HJ, Vasen HF, Barker G, Crawford G, Elliott F, Movahedi M, Pylvanainen K, Wijnen JT, Fodde R, Lynch HT, Mathers JC, Bishop DT (2012). Long-term effect of aspirin on cancer risk in carriers of hereditary colorectal cancer: an analysis from the CAPP2 randomised controlled trial. Lancet.

[15] Holmes MD, Chen WY, Li L, Hertzmark E, Spiegelman D, Hankinson SE (2010). Aspirin intake and survival after breast cancer. J Clin Oncol.

[16] Chan AT, Ogino S, Fuchs CS (2009). Aspirin use and survival after diagnosis of colorectal cancer. JAMA.

[17] Bastiaannet E, Sampieri K, Dekkers OM, de Craen AJ, van Herk-Sukel MP, Lemmens V, van den Broek CB, Coebergh JW, Herings RM, van de Velde CJ, Fodde R, Liefers GJ (2012). Use of Aspirin postdiagnosis improves survival for colon cancer patients. Br J Cancer.

[18] McCowan C, Munro AJ, Donnan PT, Steele RJC (2012). Use of aspirin post-diagnosis in a cohort of patients with colorectal cancer and its association with all-cause and colorectal cancer specific mortality. Eur J Cancer.

[19] Zaorsky NG, Buyyounouski MK, Li T, Horwitz EM (2012). Aspirin and statin nonuse associated with early biochemical failure after prostate radiation therapy. Int J Radiat Oncol Biol Phys.

[20] Choe KS, Correa D, Jani AB, Liauw SL (2010). The use of anticoagulants improves biochemical control of localized prostate cancer treated with radiotherapy. Cancer.

[21] Liu J-F, Jamieson GG, Wu T-C, Zhu G-J, Drew PA (2009). A preliminary study on the postoperative survival of patients given aspirin after resection for squamous cell carcinoma of the esophagus or adenocarcinoma of the cardia. Ann Surg Oncol.

[22] US Preventative Services Task Force (2007). Routine aspirin or non-steroidal anti-inflammatory drugs for the prevention of colorectal cancer: U.S. Preventive Services Task Force recommendation statement. Ann Intern Med.

[23] Cook N (2012). Aspirin and cancer: Evidence from randomised trials. http://www.ncri.org.uk/ncriconference/2012abstracts/mobile/abstracts/Para89.html.

[24] Bosetti C, Rosato V, Gallus S, Cuzick J, La Vecchia C (2012). Aspirin and cancer risk: a quantitative review to 2011. Ann Oncol.

[25] Cole BF, Logan RF, Halabi S, Benamouzig R, Sandler RS, Grainge MJ, Chaussade S, Baron JA (2009). Aspirin for the chemoprevention of colorectal adenomas: meta-analysis of the randomised trials. J Natl Cancer Ins.

[26] Langley RE, Burdett S, Tierney JF, Cafferty F, Parmar MK, Venning G (2011). Aspirin and cancer: has aspirin been overlooked as an adjuvant therapy?. Br J Cancer.

[27] Reader J, Holt D, Fulton A (2011). Prostaglandin E2 EP receptors as therapeutic targets in breast cancer. Cancer Metastasis Rev.

[28] Thun MJ, Jacobs EJ, Patrono C (2012). The role of aspirin in cancer prevention. Nat Rev Clin Oncol.

[29] Honn KV, Tang DG, Crissman JD (1992). Platelets and cancer metastasis: a causal relationship?. Cancer Metastasis Rev.

[30] Gupta GP, Massague J (2004). Platelets and metastasis revisited: a novel fatty link. J Clin Invest.

[31] Labelle M, Begum S, Hynes RO (2011). Direct signaling between platelets and cancer cells induces an epithelial-mesenchymal-like transition and promotes metastasis. Cancer Cell.

[32] Kopp E, Ghosh S (1994). Inhibition of NF-kappa B by sodium salicylate and aspirin. Science.

[33] Steinbach G, Lynch PM, Phillips RK, Wallace MH, Hawk E, Gordon GB, Wakabayashi N, Saunders B, Shen Y, Fujimura T, Su LK, Levin B (2000). The effect of celecoxib, a cyclooxygenase-2 inhibitor, in familial adenomatous polyposis. N Engl J Med.

[34] Thun MJ, Henley SJ, Patrono C (2002). Nonsteroidal anti-inflammatory drugs as anticancer agents: mechanistic, pharmacologic, and clinical issues. J Natl Cancer Inst.

[35] Koki AT, Masferrer JL (2002). Celecoxib: a specific COX-2 inhibitor with anticancer properties. Cancer Control.

[36] Brown JR, DuBois RN (2005). COX-2: a molecular target for colorectal cancer prevention. J Clin Oncol.

[37] Tiano HF, Loftin CD, Akunda J, Lee CA, Spalding J, Sessoms A, Dunson DB, Rogan EG, Morham SG, Smart RC, Langenbach R (2002). Deficiency of either cyclooxygenase (COX)-1 or COX-2 alters epidermal differentiation and reduces mouse skin tumorigenesis. Cancer Res.

[38] Langenbach R, Loftin CD, Lee C, Tiano H (1999). Cyclooxygenase-deficient mice. A summary of their characteristics and susceptibilities to inflammation and carcinogenesis. Ann N Y Acad Sci.

[39] Chulada PC, Thompson MB, Mahler JF, Doyle CM, Gaul BW, Lee C, Tiano HF, Morham SG, Smithies O, Langenbach R (2000). Genetic disruption of Ptgs-1, as well as Ptgs-2, reduces intestinal tumorigenesis in Min mice. Cancer Res.

[40] Liao X, Lochhead P, Nishihara R, Morikawa T, Kuchiba A, Yamauchi M, Imamura Y, Qian ZR, Baba Y, Shima K, Sun R, Nosho K, Meyerhardt JA, Giovannucci E, Fuchs CS, Chan AT, Ogino S (2012). Aspirin use, tumor PIK3C Amutation, and colorectal-cancer survival. New England Journal of Medicine.

[41] James ND, Sydes MR, Mason MD, Clarke NW, Anderson J, Dearnaley DP, Dwyer J, Jovic G, Ritchie AW, Russell JM, Sanders K, Thalmann GN, Bertelli G, Birtle AJ, O’Sullivan JM, Protheroe A, Sheehan D, Srihari N, Parmar MK (2012). Celecoxib plus hormone therapy versus hormone therapy alone for hormone-sensitive prostate cancer: first results from the STAMPEDE multiarm, multistage, randomised controlled trial. Lancet Oncol.

[42] Baigent C, Blackwell L, Collins R, Emberson J, Godwin J, Peto R, Buring J, Hennekens C, Kearney P, Meade T, Patrono C, Roncaglioni MC, Zanchetti A (2009). Aspirin in the primary and secondary prevention of vascular disease: collaborative meta-analysis of individual participant data from randomised trials. Lancet.

